# Increasing the treatment motivation of patients with somatic symptom disorder: applying the URICA-S scale

**DOI:** 10.1186/s12888-017-1400-5

**Published:** 2017-07-03

**Authors:** Johannes Mander, Georg Schaller, Hinrich Bents, Ulrike Dinger, Stephan Zipfel, Florian Junne

**Affiliations:** 1 Center for Psychological Psychotherapy, University of Heidelberg, Heidelberg, Germany; 2 Department of Psychosomatic Medicine and Psychotherapy, Medical University Hospital of Tübingen, Tübingen, Germany; 3 Department of Psychosomatic Medicine and Psychotherapy, Medical University Hospital of Heidelberg, Heidelberg, Germany; 4 Bergheimer Str. 58a, 69115, Heidelberg, Germany

## Abstract

**Background:**

Therapeutic intervention programs for somatic symptom disorder (SSD) show only small-to-moderate effect sizes. These effects are partly explained by the motivational problems of SSD patients. Hence, fostering treatment motivation could increase treatment success. One central aspect in SSD patients might be damage to motivation because of symptomatic relapses. Consequently, the aim of the present study was to investigate associations between motivational relapse struggle and therapeutic outcome in SSD patients.

**Methods:**

We assessed 84 inpatients diagnosed with SSD in the early, middle and late stages of their inpatient treatment. The *maintenance* subscale of the University of Rhode Island Change Assessment-Short (URICA-S) was applied as a measure to assess motivational relapse struggle. Additionally, patients completed measures of treatment outcome that focus on clinical symptoms, stress levels and interpersonal functioning.

**Results:**

The results from multiple regression analyses indicate that higher URICA-S *maintenance* scores assessed in early stages of inpatient treatment were related to more negative treatment outcomes in SSD patients.

**Conclusions:**

SSD patients with ambivalent treatment motivation may fail in their struggle against relapse over the course of therapy. The URICA-S *maintenance* score assessed at therapy admission facilitated early identification of SSD patients who are at greater risk of relapse. Future studies should incorporate randomized controlled trials to investigate whether this subgroup could benefit from motivational interventions that address relapse.

**Electronic supplementary material:**

The online version of this article (doi:10.1186/s12888-017-1400-5) contains supplementary material, which is available to authorized users.

## Background

Somatic symptom disorder (SSD) is characterized by physical symptoms that cannot be fully explained medically. The prevalence rates of SSD are high, ranging from 5 to 7% of the population [1]. Longitudinal studies have demonstrated significant chronicity, with up to 90% of cases lasting longer than 5 years [2, 3]. SSD symptoms are often associated with severe impairments in the general functioning of everyday life, substantial psychosocial disabilities and diminished quality of life [3, 4]. These patients show disproportionately elevated rates of medical care utilization and account for at least 25% of all doctor visits in primary care [5, 6], leading to high health care costs [7]. Consequently, psychotherapeutic treatments are crucial [8]. However, recent meta-analyses have demonstrated that therapeutic interventions for SSD patients yield only small-to-moderate effect sizes [7, 8]. Therefore, a better understanding of individual patient needs is important, as this could help to develop better treatments [9]. One core struggle among SSD patients is their highly ambivalent motivation towards participation in psychological treatments due to their usually somatically focused health beliefs [7]. More specifically, patients often search for the correct medical treatment for their problem [5], which can negatively impact treatment motivation for psychological interventions [10]. A better understanding of the motivation to change is thus critical to improving psychotherapeutic treatments for SSD [8].

The transtheoretical model (TTM; [11, 12]) offers a comprehensive theoretical framework for treatment motivation and has been investigated empirically in several studies [13]. The TTM is based on an integrative analysis of theories of behavior change from the most influential therapy schools [11] as well as on empirical studies on motivational change cycles of self-helpers with different health behaviors (e.g., smoking cessation) [14]. The model has been further improved by analyzing treatment motivation in psychotherapy patients [13], which suggests that therapeutic interventions should be adapted to match the motivational stage of the individual patient [11]. Based on the aforementioned empirical findings, the TTM distinguishes five motivational stages of change: In the *precontemplation* stage, patients have no desire for therapeutic change. In the *contemplation* stage, ambivalence about therapeutic change is central. In the *preparation stage*, there is commitment to therapeutic change. In the *action* stage, the focus is on actively working on therapeutic change, and in the *maintenance* stage, patients focus on relapse prevention.

It is of relevance to note that *maintenance* does not imply that patients have already passed through psychotherapy. Instead, it implies they have relapse struggles when trying to solve their problems. This applies to both the time in psychotherapy and the time before entering psychotherapy [11, 13]. According to the TTM, patients move through the stages of change in a spiral pattern. More specifically, this means that relapses are considered to be integral parts in the change cycle [13]. Recycling to an earlier stage of change provides patients with the opportunity to use different, more effective change strategies [11]. Further, the stages of change are operationalized using a dimensional approach [11]. This dimensional approach conceptualizes the stages of change as a multidimensional construct in which each patient has specific values in all the stages of change at the same measuring time [15]. The most widely applied dimensional measure for assessing the stages of change is the University of Rhode Island Change Assessment (URICA, [16, 17]). The URICA consists of 32 items that include eight items for each of the four subscales, *precontemplation*, *contemplation*, *action* and *maintenance*. A meta-analysis identified a medium effect size of d = .46 to describe the predictive effects of the stages of change, as measured by the URICA, on therapeutic outcome [13]. The URICA-S, a 16-item short version with excellent psychometric properties, has also been validated recently [18, 19]. It should be noted that the URICA-S *maintenance* scale includes several items that characterize struggles with relapse as being a consequence of ambivalent treatment motivation [18, 20].

Given the lack of empirical evidence on the motivational change stages in SSD using the URICA-S, research findings from anorexia nervosa (AN) patient groups may provide ideas about how to investigate the stages of change in SSD. In AN patients, for instance, the URICA-S *maintenance* scores at the beginning of treatment were helpful in identifying patients who experienced less symptom change across the course of inpatient therapy [21]. Consequently, the authors concluded that patients with higher scores on the *maintenance* domain of the URICA-S could be a subgroup in need of additional treatment elements to combat ambivalence and prevent relapse. In fact, initial empirical evidence suggests that this AN subgroup benefits from additional treatment components, such as emotion regulation and skills training related to drawbacks, additional psychoeducation, as well as aftercare programs following inpatient therapy [22].

These findings from the AN patient groups could potentially apply to SSD patients as well because relapse struggle is a typical phenomenon in those patients too [5, 10]. More specifically, many SSD patients have a long history of medical treatment before entering psychotherapy [2, 3]. These treatments sometimes result in short-term success, followed by a relapse of the problems [10]. These mostly unsuccessful attempts to address their problems before entering psychotherapy can be described in the spiral pattern of the TTM: some SSD patients try to resolve their problem (e.g., with medical treatments), have some short-term success, but then fail to maintain success and consequently relapse [10, 18]. This can also often be related to feelings of frustration and, subsequently, to decreased motivation in treatment [9]. This subgroup of SSD patients might need additional treatment components, such as interventions pertaining to emotion regulation strategies and skills training related to drawbacks, all of which could help these individuals cope with their relapse struggle. Therefore, it would be helpful to develop a stages of change measure that could facilitate the early identification of SSD patients who are at risk of suffering from motivational and relapse difficulties. For instance, although the clinical relevance of the Pain Stages of Change Questionnaire (PSOCQ; [23, 24]), a disorder-specific stages of change measure for SSD patients, has been established [10, 25], this measure has no subscale that directly assesses relapse struggle (detailed discussion in: [18]). The PSOCQ *maintenance* score reflects the ability to maintain progress [10, 22]. Hence, the URICA-S *maintenance* scale could help identify subgroups of SSD patients who need special therapeutic attention.

As mentioned above, similar to the initial empirical evidence obtained in a sample of AN patients [22], SSD patients with high URICA-S *maintenance* scores might need specific treatment elements to address motivational relapse struggle. As such, the URICA-S *maintenance* score could be applied as one component of a relapse identification tool for SSD patients. More specifically, it could be applied to the identification of subgroups of SSD patients who could specifically benefit from motivational interventions that address relapse. However, we must first obtain empirical evidence that the URICA-S *maintenance* scale can indeed identify SSD patients who are at risk of relapse. In this context, future studies should examine the extent to which therapeutic treatment can be improved using the URICA-S in SSD patients. The purpose of the current study, therefore, was to address this research gap. Based on the results of Mander et al. [21] in AN patients, we hypothesized that higher *maintenance* scores in the early, middle and late stages of therapy would be associated with more negative treatment outcomes in SSD inpatients. We specifically focused on three different relevant outcomes for SSD patients: general clinical symptoms, general stress level and interpersonal functioning.

## Method

### Treatment and study design

All patients completed an inpatient psychotherapy program at the Department of Psychosomatic Medicine and Psychotherapy of Tuebingen University, Germany. The treatment consisted of individual therapy, group therapy, art therapy and music therapy twice per week. Psychotherapy incorporated a cognitive-behavioral approach with supplementary interpersonal psychotherapeutic elements. Eighteen psychotherapists (15 female) with at least 1 year of experience conducted therapy. Individual therapy was conducted two times per week. Inpatient treatment ranged from a minimum of 14 days to a maximum of 64 days, with a mean of 38.1 (SD 14.2) days.

Patients were assessed at baseline (t_1_), after the 8th session (t_2_) and after the last session (t_3_). Treatment motivation was assessed at all three time points using the URICA-S [16, 18]. Clinical outcome measures were administered at baseline and after treatment. More specifically, at t_1_ and t_3_, patients completed the Symptom-Checklist-90-Revised (SCL-90-R; [26]), Perceived Stress Questionnaire (PSQ; [27]), and Inventory of Interpersonal Problems (IIP; [28]). Additionally, the Structured Clinical Interview, German version (SCID-I, [29, 30]), was administered to all patients at baseline. The initial SCID-I assessment was conducted by one of two PhD-students who had previously completed a university-based SCID training. A university-affiliated expert regularly supervised these students. The local ethics committee of the medical faculty at the University of Tuebingen approved the study protocol (project number 29/2009B02) in compliance with the Helsinki Declaration. All participants provided written informed consent prior to study participation.

### Subjects

Eighty-four inpatients being treated for SSD participated in the study. To be included, subjects had to have received an SCID-I primary diagnosis of a full syndrome of SSD. The general exclusion criteria were as follows: (1) patient age younger than 18 years or older than 59 years, (2) insufficient German language skills, (3) psychotic or substance-related disorders as comorbidities, and (4) current suicidal risk. Because we intended to investigate a naturalistic sample, comorbidities with mental disorders not on the exclusion list were not considered to be exclusions for study participation if SSD was the main treatment diagnosis. Overall, the study sample was 71.4% female. The average age of participants was 48.0 years (SD 11.9), while 59.5% were married/with partner, 19% had an A-level degree and 88.2% had a formal professional qualification. Furthermore, 50.0% were employed, 4.8% were still in job training, 25% were retired and 20.2% were unemployed. The sample included 59.5% with a diagnosis of major depression (36.9% single episode, 22.6% recurrent depression) and 10.7% with an anxiety disorder (9.5% panic disorder and agoraphobia; 1.2% specific phobia) as a comorbidity. According to patient interviews and medical data, all patients suffered from SSD symptoms for several years.

### Measures

#### URICA-S

The URICA-S [18] is a short version of the URICA [16]. The URICA-S consists of 16 items that are rated on a scale from 0 (does not apply) to 4 (fully applies) and measures four stages of change (*precontemplation*, *contemplation*, *action*, *maintenance*) with 4 items per subscale (items listed in the appendix). In a sample of 253 patients, Mander et al. [18] demonstrated an excellent factor structure with factor loadings of .52 ≤ λ ≤ .87. Confirmatory factor analyses supported the exploratory model. The instrument yielded acceptable-to-excellent internal consistencies, with .61 ≤ α ≤ .84, along with high correlations with the subscales of the original long-form URICA, with .83 ≤ *r* ≤ .96. The *contemplation*, *action* and *maintenance* subscales and *committed action* global score significantly predicted therapeutic outcome. The *maintenance* score from the URICA-S specifically refers to the struggle with relapse, with stronger negative values indicative of more problems with relapse. Hence, it might be especially important to identify patients at risk of further chronicity. All items of the URICA-S are listed in the online supplement Additional file 1.

#### SCL-90-R

The SCL-90-R [26, 31] is a measure of general symptom severity. It consists of eleven subscales totaling 90 items rated along a 5-point scale. The measure has demonstrated excellent internal consistencies, with .79 ≤ α ≤ .89, and good retest-reliabilities, with .69 ≤ *r* ≤ .92. Acceptable construct validity has also been reported, with scale-outcome correlations between .27 ≤ *r* ≤ .81.

#### PSQ

The PSQ [27, 32] is a 30-item scale that was designed to evaluate the stress level of a patient within the seven factors of *harassment*, *overload*, *irritability*, *lack of joy*, *fatigue*, *worries,* and *tension.* Each item is rated along a 5-point scale. The PSQ has demonstrated excellent psychometric properties, with internal consistencies of .80 ≤ α ≤ .86, as well as convergent validity via correlations of .56 ≤ *r* ≤ .73 with other instruments referring to stress, anxiety, and depression. It has further demonstrated sensitivity to change across the course of therapy.

#### IIP

The IIP is a 64-item instrument that utilizes a circumplex structure to assess interpersonal problems [28, 33, 34] . Its eight scales include *domineering*, *intrusive*, *overly nurturant*, *exploitable*, *nonassertive*, *socially avoidant*, *cold*, and *vindictive,* rated on a 5-step scale. The IIP has shown excellent psychometric properties, with .75 ≤ α ≤ .94, and criterion validity has been demonstrated through correlations with the SCL-90-R, with .07 ≤ *r* ≤ .75.

### Statistical analyses

To identify empirical associations between the stages of change and therapeutic outcomes, we initially calculated a set of correlation analyses, computing separate analyses for each of the three therapeutic outcome measures. More specifically, we correlated the URICA-S subscales with the SCL-90-R, PSQ, and IIP global scores in separate analyses. In the next step, we calculated a set of hierarchical regression analyses, computing separate regression analyses for each of the three therapeutic outcome measures as the dependent variable. More specifically, we analyzed one regression model with the SCL-90-R global score at t_3_, one with the PSQ global score at t_3_ and one with the IIP global score at t_3_ as the dependent variable. In each model, we blockwise entered the four URICA-S subscale scores as well as the baseline score of the relevant outcome measure as predictors. In the first block, we entered the global score of the relevant outcome measure at baseline as an autoregressor. In the second block, we always entered the *maintenance* score because it demonstrated the strongest association with the therapeutic outcome in the correlation analyses. The order in which the other subscales were entered in subsequent blocks depended on the strengths of their correlations with the outcome measures.

## Results

Correlation analyses of the URICA-S revealed the following results for the stages of change subscales: *Precontemplation* was not significantly associated with any of the therapeutic outcome scores. *Contemplation* at t_1_ was significantly associated with the PSQ, but no other significant associations emerged for *contemplation*. *Action* was not significantly associated with any of the therapeutic outcome scores. M*aintenance* was significantly associated with all three outcome measures (the SCL-90-R, the PSQ and the IIP) at all three measuring times. The results of the correlation analyses are depicted in Table 1.

**Table 1:** Correlations of the URICA subscales at all three measuring times with SCL, PSQ and IIP at the end of therapy

	Precontemplation	Contemplation	Action	Maintenance
SCL-90**-**R				
t_1_	.11	.15	.03	.48^**^
t_2_	.13	.12	−.07	.38^**^
t_3_	.00	.22	−.04	.43^**^
PSQ				
t_1_	.08	.28^*^	.19	.48^**^
t_2_	.02	−.05	−.34	.34^**^
t_3_	.15	.17	−.03	.38^**^
IIP				
t_1_	.11	.18	.06	.42^**^
t_2_	.07	.00	−.04	.32^*^
t_3_	−.04	.09	−.14	.30^*^

t_1_/t_2_/t_3_ = at baseline/8th/last therapy session

*SCL-90-R* Symptom-Checklist-90-Revised, *PSQ* Perceived Stress Questionnaire, *IIP* Inventory of Interpersonal Problems

^*^ = *p* < .05; ^**^ = *p* < .01

In the multiple regression analyses, only *maintenance* proved to be a significant predictor of outcome when controlling for the baseline outcome scores.

Concerning SCL-90-R, *maintenance* at t_1_ was a significant predictor of SCL-90-R at t_3_ while controlling for SCL-90-R at t_1_, ∆*R* ^2^ = .08, *p* = .005. *Maintenance* at t_2_ and t_3_ did not significantly predict SCL-90-R at t_3_. However, trends (*p* < .10) were observable, with ∆*R* ^2^ = .05. *p* = .065 for *maintenance* at t_2_ and ∆*R* ^2^ = .022, *p* = .069 for *maintenance* at t_3_.

Concerning the PSQ, *maintenance* was a significant predictor at all three measuring times. More specifically, the *maintenance* score was a significant predictor of the PSQ at t_3_ while controlling for the PSQ at t_1_; with ∆*R* ^2^ = .08, *p* < .001 for *maintenance* at t_1_; ∆*R* ^2^ = .06, *p* = .022 for *maintenance* at t_2_; and ∆*R* ^2^ = .07, *p* = .017 for *maintenance* at t_3_.

Concerning the IIP, *maintenance* at t_1_ and t_2_ were significant predictors. More specifically, the *maintenance* score was a significant predictor of the IIP at t_3_ while controlling for the IIP at t_1_, with ∆*R* ^2^ = .02, *p* = .023 for *maintenance* at t_1_; and with ∆*R* ^2^ = .03, *p* = .034 for *maintenance* at t_2_. Maintenance at t_3_ was not a significant predictor of the IIP at t_3_. However, a trend (*p* < .10) was observable, with ∆*R* ^2^ = .02, *p* = .087.

None of the other stages of change proved to be a significant predictor in any regression model. For parsimonious reasons, we only report the significant regression models in Tables 2, 3 and 4.

**Table 2:** Significant linear regression coefficients of stages of change predicting SCL-90 symptomatology at the end of therapy

						95% CI		
	B	SE B	β	t	*p*	Lower bound	Upper bound	R^2^
Model 1: Maintenance t_1_								.57
Constant	.31	.15		2.11	.039	.02	.61	
SCL-90 baseline	.26	.08	.36	3.31	<.001	.10	.42	
Maintenance t_1_	.23	.08	.32	2.92	.005	.07	.38	
Model 2: Maintenance t_2_								.53
Constant	.36	.16		2.27	.027	.04	.69	
SCL-90 baseline	.25	.07	.42	3.50	<.001	.11	.39	
Maintenance t_2_	.15	.08	.23	1.89	.065	−.01	.31	
Model 3: Maintenance t_3_								.70
Constant	−.18	.13		−1.33	.191	−.45	.09	
SCL-90 baseline	.84	.09	.77	9.14	<.001	.66	1.03	
Maintenance t_3_	.10	.05	.16	1.90	.069	−.01	.21	

t_1_/t_2_/t_3_ = at baseline/after 8th therapy session/ end of therapy. Model 1 / Model 2 / Model 3 = significant stages of change predictors at baseline/8th/last therapy session for SCL-90 at the end of therapy.

*SCL-90-R* Symptom-Checklist-90-Revised

**Table 3:** Significant linear regression coefficients of stages of change predicting PSQ symptomatology at the end of therapy

						95% CI		
	B	SE B	β	t	*p*	Lower bound	Upper bound	R^2^
Model 1: Maintenance t_1_								.49
Constant	.06	.06		.92	.362	−.07	.18	
PSQ baseline	.61	.11	.52	5.68	<.001	.40	.83	
Maintenance t_1_	.05	.02	.31	3.33	<.001	.02	.08	
Model 2: Maintenance t_2_								.43
Constant	.03	.07		.33	.74	−.12	.17	
PSQ baseline	.67	.12	.57	5.60	<.001	.43	.91	
Maintenance t_2_	.04	.02	.24	2.35	.022	.006	.08	
Model 3: Maintenance t_3_								.42
Constant	.02	.08		.26	.79	−.15	.19	
PSQ baseline	.67	.14	.54	4.94	<.001	.40	.95	
Maintenance t_3_	.04	.02	.27	2.46	.017	.01	.08	

t_1_/t_2_/t_3_ = at baseline/after 8th therapy session/ end of therapy. Model 1 / Model 2 / Model 3 = significant stages of change predictors at baseline/8th/last therapy session for PSQ at the end of therapy.

*PSQ* Perceived Stress Questionnaire

**Table 4:** Significant linear regression coefficients of stages of change predicting IIP symptomatology at the end of therapy

						95% CI		
	B	SE B	β	t	*p*	Lower bound	Upper bound	R^2^
Model 1: Maintenance t_1_								.68
Constant	.08	.12		.71	.48	−.15	.32	
IIP baseline	.85	.08	.75	10.53	<.001	.69	1.01	
Maintenance t_1_	.09	.04	.17	2.33	.023	.01	.17	
Model 2: Maintenance t_2_								.66
Constant	.00	.15		.01	.996	−.30	.30	
IIP baseline	.88	.09	.76	9.67	<.001	.70	1.07	
Maintenance t_2_	.09	.04	.17	2.18	.034	.01	.18	
Model 3: Maintenance t_3_								.64
Constant	.11	.16		.68	.501	−.21	.43	
IIP baseline	.86	.10	.76	8.78	<.001	.66	1.05	
Maintenance t_3_	.07	.04	.15	1.75	.087	−.01	.16	

t_1_/t_2_/t_3_ = at baseline/after 8th therapy session/ end of therapy. Model 1 / Model 2 / Model 3 = significant stages of change predictors at baseline/8th/last therapy session for IIP at the end of therapy.

*IIP* Inventory of Interpersonal Problems

## Discussion

SSD patients show disproportionately elevated rates of medical care utilization, leading to high health care costs [7]. Further, therapeutic intervention programs for SSD patients show only small-to-moderate effect sizes [7, 8]. This is partly explained by the ambivalent motivation of SSD patients to receive psychological treatments [10]. Hence, fostering treatment motivation could increase treatment success in this patient group [8]. The aim of the present study was to investigate associations between the TTM stages of change and general symptomatology and general stress level, as well as interpersonal functioning in SSD patients. We specifically hypothesized that higher scores on the URICA-S *maintenance* scale, as an indicator of motivational relapse struggle, would be associated with more negative treatment outcomes in SSD inpatients. Of note, our results indicate that URICA-S *maintenance* at the first measuring time was a significant predictor of all three outcome measures. This applied to both the correlation analyses and longitudinal multiple regression analyses controlling for baseline symptom severity. These findings indicate that the higher the *maintenance* score at the beginning of therapy, that is, the greater the struggle with relapse, the lower the change in the three outcome measures across the course of therapy. This is clinically relevant, as it indicates that assessment of the patients´ *maintenance* stage of change at the beginning of therapy can help identify patients who will be more likely to have negative treatment outcomes. Furthermore, URICA-S *maintenance* at therapy admission significantly predicted patients’ general clinical symptoms, general stress levels and interpersonal functioning. Consequently, the results indicate that URICA-S *maintenance*, as assessed early in treatment, is an important predictor of three essential aspects of the general functioning of SSD patients.

However, an important limitation of our results is that *maintenance* assessed at t_2_ and t_3_ did not significantly predict general symptom severity as assessed by the SCL-90-R or interpersonal functioning as assessed by the IIP. Here, only trends (*p* < .10) toward predictive effects were observable. These effects could possibly reach significance with more statistical power in larger SSD samples. However, identifying this aspect empirically remains a task for future research. Hence, given our current research results, it remains unclear whether *maintenance* assessed later in therapy is still a relevant predictor of the therapeutic outcome. Consequently, our results must be regarded as preliminary and should be interpreted with caution. Further, and similar to our previous findings in AN patients [21], no other stages of change were significant predictors of outcome.

Hence, in SSD patients, the *maintenance* score assessed at therapy admission seems to be an indicator of the relapse threat, which could be helpful for early identification of inpatients who could improve during therapy as well as those who might suffer from repeated drawbacks. In many SSD patients, the course of their disorder is characterized by high chronicity and recurrent relapses, while the first months after inpatient treatment seem to represent a highly vulnerable phase [7, 8]. Consequently, should our results be replicated in future studies, the URICA-S *maintenance* ratings could be used to supplement the clinical judgments of expert diagnosticians to identify high-risk patient subgroups that need further treatment elements in addition to routine therapy. More specifically, similar to preliminary studies in AN patients [22], these patient subgroups could receive a specific treatment to address ambivalence, skills training related to drawbacks, and additional psychoeducation as well as aftercare programs following inpatient treatment.

As mentioned previously, the PSOCQ can also be a disorder-specific stage of change measure for SSD patients, and several studies have documented its clinical utility (e.g. [10, 24]). Future research should use the PSOCQ to examine the extent to which our current study results can be replicated with a disorder-specific measure. In contrast to the URICA-S, where *maintenance* refers to relapse struggle, the PSOCQ *maintenance* scale reflects the ability to maintain progress [23, 24]. The current study results indicate that a relapse measure is important in SSD research, as high chronicity and recurrent relapses are important characteristics of the disorder [7, 8]. Consequently, it might be of interest to devise a new, more differentiated disorder-specific *maintenance* scale for the PSOCQ that contains a progress maintenance component and relapse facet.

The current study has the following limitations: First, our study was conducted in an inpatient setting. Hence, the generalizability of the effects to other treatment setting remains unclear and should be addressed in future studies. Second, we conducted a naturalistic study without a control group. Future research should try to directly implement motivational interventions to improve the outcomes of patients who score high for URICA-S *maintenance* at the beginning of therapy. Consequently, a randomized controlled design, such as one that incorporates motivational interviewing [35] in the experimental condition and typical treatment in the control condition, should be applied. Third, two predictors in regression analyses were only marginally significant. Hence, our results should be interpreted with caution. However, when interpreting the results, not only should the specific effect of the single predictor be recognized but the whole pattern of results should be interpreted. More specifically, *maintenance* was a significant predictor of therapeutic outcome irrespective of measuring time and whether general clinical symptoms, the general stress level or interpersonal functioning were entered as dependent variables. Consequently, *maintenance* seems to be one important aspect that should be addressed to increase the chance of successful psychotherapy. Fourth, in the current study, approximately 60% of patients suffered from comorbid depressive disorders. SSD patients with and without depression might show different patterns regarding the effects of treatment motivation on the therapeutic outcome. In our study, the statistical power to identify subgroup effects of SSD patients with and without comorbidities was too low. Hence, future studies should investigate this aspect in more adequately powered subsamples. Finally, future studies should investigate associations between motivation and outcome on a session-to-session basis, as this could capture greater variations and nuances within the therapeutic process.

## Conclusions

In conclusion, the present study analyzed associations between the stages of change assessed with the URICA-S and therapeutic outcome in SSD patients. The URICA-S *maintenance* score assessed at the beginning of therapy could be used to help identify SSD patients who have a relapse risk. Future studies should incorporate randomized controlled trials to investigate whether this subgroup could benefit from motivational interventions to address relapse.

## Additional file

Additional file 1: Items_of_the_URICA-S, the additional file includes all items of the URICA-S scale. (DOCX 12 kb)
