# Supplementary material for: Increasing the treatment motivation of patients with somatic symptom disorder: applying the URICA-S scale
Source: BMC Psychiatry. 2017 Jul 3;17:240. doi: 10.1186/s12888-017-1400-5 (PMC5496251; doi:10.1186/s12888-017-1400-5)
Supplement: Additional file 1: — Items_of_the_URICA-S, the additional file includes all items of the URICA-S scale. (DOCX 12 kb) [file 12888_2017_1400_MOESM1_ESM.docx]

**Items of the URICA-S**

Itemnumbers in brackets

**Precontemplation**

I'm not the problem one. It doesn't make much sense for me to be here. (1)

I guess I have faults, but there's nothing that I really need to change. (5)

All this talk about psychology is boring. Why can't people just forget about their

problems? (9)

I have worries but so does the next guy. Why spend time thinking about them? (13)

**Contemplation**

I'm hoping this place will help me to better understand myself. (2)

I wish I had more ideas on how to solve the problem. (6)

Maybe this place will be able to help me. (10)

I hope that someone here will have some good advice for me. (14)

**Action**

I am doing something about the problems that had been bothering me. (3)

At times my problem is difficult, but I'm working on it. (7)

I am really working hard to change. (11)

I am actively working on my problem. (15)

**Maintenance**

It worries me that I might slip back on a problem I have already changed, so I am here to seek help. (4)

I'm not following through with what I had already changed as well as I hoped, and I'm here to prevent a relapse of the problem. (8)

I'm here to prevent myself from having a relapse of my problem. (12)

It is frustrating, but I feel I might be having a recurrence of a problem I thought I had resolved. (16)
